# Activators of the 26S proteasome when protein degradation increases

**DOI:** 10.1038/s12276-024-01385-x

**Published:** 2025-01-08

**Authors:** Donghoon Lee

**Affiliations:** 1 Department of Cell Biology, Harvard Medical School, 240 Longwood Avenue, Boston, MA 02115 USA; 2 Present Address: Biology, College of Science, Mathematics and Technology, Wenzhou-Kean University. 88 Daxue Road, 325060 Wenzhou, Zhejiang China

**Keywords:** Proteasome, Ubiquitylation

## Abstract

In response to extra- and intracellular stimuli that constantly challenge and disturb the proteome, cells rapidly change their proteolytic capacity to maintain proteostasis. Failure of such efforts often becomes a major cause of diseases or is associated with exacerbation. Increase in protein breakdown occurs at multiple steps in the ubiquitin-proteasome system, and the regulation of ubiquitination has been extensively studied. However, the activities of the 26S proteasome are also stimulated, especially under highly catabolic conditions such as those associated with atrophying skeletal muscle, proteotoxic stress such as heat shock and arsenite, or hormonal cues such as cAMP or cGMP agonists. Among the proteins that enhance proteasomal degradation are the PKA, PKG, UBL-UBA proteins and the Zn finger AN1-type domain (ZFAND) family proteins. ZFAND proteins are of particular interest because of their inducible expression in response to various stimuli and their abilities to control protein quality by stimulating the 26S proteasome and p97/VCP. The regulatory roles of ZFAND proteins appear to be important not only for the control of protein degradation but also for other cellular processes, such as mRNA stability and signaling pathways. This review summarizes the known functions of proteasome activators and discusses their possible roles in regulating proteostasis and other cellular processes.

## ZFAND proteins enhance proteasome activity and proteostasis

For many years, scientists have studied how cells break down proteins using a proteolytic complex like the proteasome. The proteasome is a complex machine that degrades majority of intracellular proteins. However, its activation mechanism under stresses is not fully understood. Researchers explored proteins called ZFANDs, which can activate the proteasome. The studies used various methods to understand how ZFAND proteins work and found that ZFAND5, as an example, helps the proteasome break down proteins more efficiently. This is important because it can help cells manage stresses by removing unwanted proteins. The results showed that ZFAND5 binds to the proteasome and enhances its activity, especially in stresses causing muscle wasting. This discovery helps us understand how cells adapt to stresses and could lead to new treatments for diseases where protein breakdown is disrupted.

This summary was initially drafted using artificial intelligence, then revised and fact-checked by the author.

## Introduction

For decades, numerous elegant studies have demonstrated how cellular proteins are degraded in precisely controlled manners by chaperones, autophagy and the ubiquitin-proteasome system (UPS)^1^. In mammalian cells growing under normal culture conditions, 26S proteasomes degrade more than 80% of proteins, whereas this proteolytic capacity is altered in different cellular contexts^2^. To achieve highly selective and specific ubiquitination of proteins, ubiquitination is controlled by many factors, such as specific types of enzymes, the length of ubiquitination, deubiquitnases, and posttranslational modifications^1^. As the primary proteolytic machinery, the 26S complex captures and processes ubiquitinated proteins through multiple steps in a coordinated and controlled manner. These regulatory steps include recognition of the substrate and its binding to one of three ubiquitin-interacting subunits, the induction of structural changes upon substrate binding, the commitment step by deubiquitination and unfolding by ATPase activities, and the subsequent translocation of the unfolded region of the substrate into the catalytic chamber of the 20S complex^3–6^. Moreover, remarkable details concerning the kinetics of the substrate binding modes to 26S and subsequent deubiquitination have also been demonstrated at the single-molecule level^7,8^.

The 26S proteasome is an approximately 2.4 MDa multisubunit protease complex. It consists of the 19S complex, also known as the regulatory particle (RP) or proteasome activator 700 (PA700), and the 20S complex (core particle) and contains three ubiquitin-binding subunits (Rpn1, Rpn10 and Rpn13), multiple enzymatic activities such as deubiquitinase (Rpn11), ATPases (Rpt1–6), and three peptidases (β1, β2 and β5)^3^. In addition, there are more enzymatic activities associated with the substoichiometric subunits, such as USP14/UBP6, UBE3C/HUL5, and UCH37. These extremely complex enzymatic activities are precisely coordinated to target specific proteins to the proteasome and subsequent events for hydrolysis.

Proteasomal activities are also controlled by many of its interacting proteins and post-translational modifications^3,9,10^. Among those factors, PKA, PKG and ubiquitin-like (UBL)-ubiquitin-associated (UBA) proteins are of particular interest due to their stimulatory activities on proteasome activities. In addition, there are regulators that are induced when protein breakdown increases and promote particular functions of the 26S proteasome and p97, two key components of the UPS^11–16^. In this review, the factors that promote the activities of the proteasome and p97/VCP are summarized, and the proteins containing the AN1-type Zn finger domain, changes in their expression, known functions, and their stimulatory effects on the proteasome and p97 in the adaptive response to various stimuli are discussed.

## Activation of the proteasome when proteolysis increases

Notably, a study using cryo-electron tomography demonstrated that a large population (approximately 73%) of the 26S complex remains in an inactive state (also known as the ground state) under normal conditions^17^. This study suggested that under conditions of increased proteotoxic stress caused by the accumulation of misfolded or aggregated proteins, the 26S fractions in the conformation representing the substrate-processing states increase. Indeed, the following in situ tomogram study revealed that 26S complexes in the substrate-processing conformation are enriched close to poly Gly-Ala aggregates^18^.

Overall, protein degradation increases in response to various stimuli, and the effects of several physiological conditions on proteasomal activities have been investigated^19^. For example, agents that activate the cAMP and cGMP signaling pathways increase the degradation of short- and long-lived proteins, respectively^20,21^. In in vitro assays, PKA and PKG phosphorylate proteasome subunits and increase the hydrolysis of ATP and short peptides^20,21^. Because these stimulatory agents have therapeutic benefits in cardiac failure, potentially in tauopathies and other diseases associated with protein aggregation, the activation of proteasome capacity under these conditions proposes therapeutic potential^20,22,23^. Two additional physiological conditions that cause a rapid increase in overall protein degradation are heat shock and skeletal muscle atrophy. The cellular adaptive response to heat shock protects cells from the toxicity of accumulated misfolded or damaged proteins by increasing their folding capacity or clearing toxic aggregates by increasing protein breakdown^24^. Our recent study demonstrated that heat shock activates the proteasomal degradation of ATP, short peptides and ubiquitinated substrates^25^. However, the mechanism by which the proteasome becomes more active remains to be clarified. To study protein synthesis and degradation, the skeletal muscle of rodents has been used as an in vivo model since late 1960, primarily by the Fred Goldberg laboratory^26^. Since then, numerous studies have explored intracellular protein turnover in detail, and the signaling pathways governing the control of muscle size and the primary degradation machineries have been thoroughly reviewed^27^. Our recent efforts in studying proteasome activation demonstrated that Zn finger AN1-type domain 5 (ZFAND5), the inducible gene in atrophying skeletal muscle, is a 26S proteasome activator by enhancing multiple steps of substrate processing^28,29^. There are more ZFAND family proteins that are involved in critical cellular processes. For example, these proteins regulate proteasome and p97 functions, promote cell survival upon arsenite treatment and play regulatory roles in signaling pathways, tumor invasion, and cell differentiation^11,12,16,30^. Despite the physiological significance of their roles, their mechanistic details remain largely unexplored; thus, their potential roles are suggested and discussed below.

## Phosphorylation for proteasome activation

Several subunits of both the 19S and 20S complexes undergo phosphorylation; among them, those that specifically control proteasome activity are briefly discussed below^10^. The physiological importance of proteasome activation by phosphorylation has been thoroughly covered in a recent review^19^. The activation of the proteasome by the cAMP‒PKA pathway in rat kidney cells via Rpt6 phosphorylation has been previously reported, but its physiological function is not known^31^. In rat spinal cord neurons, treatment with PKA agonists such as dibutyryl-cAMP (db-cAMP) and forskolin seem to increase the levels of 26 proteasome, p97, and the C-terminus of Hsc70-interacting protein (CHIP), suggesting that attenuation of neuronal damage is induced by the cytotoxicity of prostaglandin J2 treatment^32^. With respect to its role in protein homeostasis, proteasome activities enhanced by cAMP–PKA have been reported to increase the degradation of short-lived proteins, which seems to promote the proteolytic capacity of proteasomes compromised by tau toxicity in cortical tissues^20,23,33^. Although the stimulatory effect of cAMP-PKA results from the phosphorylation of a specific residue (S14) of Rpn6, the exact mechanism by which Rpn6-S14 phosphorylation alters proteasome activity remains to be determined.

Proteasome activation by increasing cGMP through the inhibition of PDE5 with tadalafil, the soluble guanylyl cyclase stimulator BAY41-2272, or overexpressing PKG1α enhances the breakdown of both long- and short-lived proteins^22^. Although PKG has been reported as a proteasome activator for more than ten years^34^, a target protein, e.g., the proteasome subunit or interacting protein, of PKG for activation has not yet been reported^22^. As a PKG target in the UPS under a similar context, in cardiomyocyte ischemic injury, CHIP is phosphorylated to facilitate the clearance of aggregated proteins^35^.

Proteasome activation by phosphorylation also regulates cell proliferation. Threonine 25 phosphorylation of Rpt3 by dual-specificity tyrosine kinase 2 (DYRK2) during the S and G2/M phases of the cell cycle is critical for cell division^36^. A nonphosphorylatable Thr25 mutant of Rpt3 (T25A) generated using the CRISPR/Cas9 knock-in system reduced tumor growth, which appears to be attributed to attenuated proteasome activity. Guo and colleagues reported that Rpn1 phosphorylation at Ser361 is also required for proteasome activity and cell growth, which is reversed by UBLCP1 and contributes to a cellular adaptive response to oxidative stress^37^.

Despite these intriguing functions of phosphorylated proteasomes in diverse physiological contexts, the details of the activation mechanism by which the hydrolysis of ubiquitinated substrates, short peptides or ATP is stimulated by phosphorylation remain to be investigated.

## Other posttranslational modifications for proteasome regulation

Among other modifications, ADP-ribosylation is known to stimulate the activity of the 20S proteasome upon H_2_O_2_ treatment or neuroinflammatory stimulation, such as TNF-α^38,39^. Additionally, acetylation of 20S has been reported, but further evidence, such as mutagenesis of the residues for functional relevance, is needed^40^. S-glutathionylation of 20S is also suggested to increase the number of gate-opening 20S in cells^41^. As predicted by its size and numbers of core and interacting subunits, the proteasome undergoes virtually all kinds of posttranslational modifications, which have been well listed and discussed elsewhere^10^.

## UBL-UBA proteins

UBL-UBA proteins (e.g., Rad23B, Ubiquilins, DDI2) are known to bind both proteasome and ubiquitin conjugates, which is the main feature of the shuttling factors^9^. The UBL domains of RAD23 and DSK2 specifically bind the T1 site of Rpn1, whereas another important 26S regulator, USP14, interacts with the T2 site of Rpn1 subunit^42^. This meticulous study not only revealed new ubiquitin-binding subunits of the proteasome but also provided insights into how UBL-UBA proteins contribute to proteasomal activities. Indeed, our recent findings demonstrated the stimulatory action of UBL-UBA proteins such as USP14, RAD23B, DDI2 and even PARKIN^43,44^. These studies raised interesting questions, such as the following: do they all bind intrinsic ubiquitin receptors, i.e., Rpn1, Rpn10 or Rpn13, as does Rad23^42^? Because the UBL-UBA proteins are constitutively expressed, do they compete with each other for binding to the proteasome? Do they compete with ubiquitinated substrates for binding to the proteasome and activate the proteasome? Because many UBL-UBA proteins are known to play critical roles in neurodegenerative diseases, e.g., Alzheimer’s disease and Parkinson’s disease, the regulation of their interaction with the proteasome and its substrates may present novel therapeutic applications against these diseases. The significance and physiological relevance of UBL-UBA proteins to proteasome activation have been covered in a recent review^19^.

The majority of UBL-UBA proteins are likely to function as shuttling factors for proteasomal protein breakdown. Among the key remaining questions related to protein degradation by the proteasome include the mechanism of substrate recruitment to the 26S proteasome by shuttling factors such as the Rad23 family (hHR23A, hHR23B, and scRad23), Dsk2 family (Ubiquilin 1-4 and scDsk2), Ddi1 family (hDdi1, hDdi2 and scDdi1) and ZFAND family^9^. Genetic studies have demonstrated that double mutants, e.g., Rad23 and Dsk2 or Rad23 and Ddi1, accumulate certain proteins that are likely to be clients of those shuttling factors^45–47^. However, overexpression of Rad23 stabilizes client proteins, e.g., Pds1, and the UFD substrate, which seemingly contradicts the expected stimulatory role of Rad23 in the degradation of client proteins^46,47^. However, studies on the roles of the ubiquitin-associated (UBA) domain that binds ubiquitin chains and inhibits disassembly have suggested that excessive UBA probably sequesters ubiquitinated proteins from the 26S proteasome and thus accounts for the inhibitory effect of UBA overexpression^48–50^. In addition to the inhibitory effect of UBA overexpression in cells, the results of an in vitro degradation assay in which purified 26S proteasome and Rad23 substrate degradation were reconstituted in the purified system were also inhibited by the addition of Rad23^51^. Thus, among the remaining questions concerning the functions of the shuttling factors are, why there are many shuttling factors, and are they targeting different sets of proteins for proteasomal degradation? In the proteasomal hydrolysis of substrates, how do factors recruit substrates to 26S proteasomes? What has been observed in vivo remains to be seen in vitro.

## ZFAND proteins

Currently, eight family proteins have been reported, all of which have one or two AN1-type Zn fingers (Table 1). Overall, the functions of these proteins have not been well investigated, although their potential roles in controlling important cellular processes, e.g., in response to proteotoxic stresses and increased proteolysis, have been shown. Their correlations with some diseases, known functions, and biochemical properties are listed, and some possibilities are discussed as cellular adaptation tools against various stresses.

**Table 1:** ZFAND family proteins in several organisms.

Genes	Functions	Domains^c^				Refs
Mammal		AN1	A20	UBL	UIM or Others	
ZFAND1	• Interacts with proteasome and p97 • Enhances clearance of stress granules formed by arsenite	Two	None	One		^1^
ZFAND2A	• Inducible expression by arsenite • Interacts with 26S through Rpn1 • Stimulates proteasomal peptidase activity	Two	None	None	UBZ^a^	^2,3^
ZFAND2B	• Interacts with proteasome upon exposure to arsenite • Interacts with p97 and cofactors on ER membranes by its membrane-association motif • Role in suppressing myeloproliferative neoplastic phenotypes	Two	None	None	2 UIM, 1 CAAX, 1 UBZ^a^	^2–5^
ZFAND3	• Transcription factor for gene expression linked to glioblastoma cell invasion • Interacts with proteasome	One	One	None		^6^
ZFAND4	N/A	One	None	One		UniProt
ZFAND5	• Interacts with Rpt5 and causes large conformational changes • Stimulates proteasomal degradation of short peptides, ATP, ubiquitinated proteins and the deubiquitination of substrate once on the proteasome • Colocalizes with aggresome induced by proteasome inhibition • Inducible expression by the conditions causing wasting of skeletal muscle • KO reduces muscle atrophy • Inhibits NF-κB activity • Stabilizes mRNA	One	One	None		^7–9^
ZFAND6	• Is involved in the import of peroxisomal proteins • Interacts with Pex6 and monoubiquitinated Pex5 • Involved in both the inhibition and stimulation of NF-κB	One	One	None		^73–75^
ZFAND7	• Mutations are associated with the degeneration of motor neurons or CMT2	One^a^	None	None	1 Helicase, 1 R3H	^79,83^
**Plant**^b^						
AtSAP5	• Inducible expression upon various abiotic stresses • Improves resistance to dehydration, cold and heat • AN1 domain shows ubiquitin ligase activity	One	One	None		^14^
OSISAP1	• Inducible expression upon various abiotic stresses • Overexpression enhances tolerance to dehydration, salt and cold	One^a^	One^a^	None		^15,16^
Pha13	• Overexpression improves resistance against viruses and bacteria • A20 domain shows ubiquitin ligase activity	One	One	None		^57^
**Fruit fly**						
CG12795	N/A	Two^a^	None	None		FlyBase
CG30094	N/A	One^a^	None	None		FlyBase
CG15368	N/A	One^a^	None	None		FlyBase
Doctor No	• Drosophila ortholog of ZFAND6 • Is a component of the JAK/STAT pathway • Is required for endocytic trafficking of Dome, the receptor of the pathway	One^a^	One^a^	None		^18^
***C. elegans***						
AIP-1	• Inducible expression upon exposure to arsenite • Is required for the normal lifespan • Protects animals from proteotoxicity	Two	None	None	1 UIM, 1 CAAX	^2–4^
**Yeast**						
Cuz1	• Interacts with Rpn2 and Cdc48 • Protects cells from proteotoxicity	One	None	One		^19,20^

^a^The presence of a domain is identified by sequence analysis but not functionally characterized.

^b^Only three genes are shown due to space limitations.

^c^The numbers and presence of the domains are shown based on published studies or predictions in UniProt.

### In yeast, worms, fruit flies and plants

In *Saccharomyces cerevisiae*, there seem to be two ZFAND genes, Cuz1 (Cdc48-associated UBL zinc finger protein 1)/YNL155w and YOR052C. Interestingly, because their promoters contain proteasome-associated control element (PACE), whether their transcription might be coordinately regulated with ubiquitin availability by Rpn4 remains to be investigated^13,14^. The human ortholog of Cuz1 is ZFAND1 (see below), and the presence of only the AN1 domain of YOR052C is similar to that of ZFAND2A/AIRAP (arsenite-inducible RNA-associated protein), although their sequence similarities are very low^13^. Interestingly, Cuz1 and YOR052C are required to protect cells against proteotoxic stresses, e.g., arsenite, suggesting their roles in the UPS^13,14^. Indeed, Cuz1 interacts with the proteasome by binding Rpn2 of 19S RP, supporting its role in resistance to proteotoxicity, but how YOR052C contributes to resistance remains uncharacterized^14^. Cuzl also interacts with ubiquitin and Cdc48 by its UBL, which is divergent from the ubiquitin amino acid sequence. Together with its interaction with the proteasome, these studies suggest a possible role of Cuz1 in delivering ubiquitinated substrates to Cdc48 or the proteasome or substrates unfolded by Cdc48 to the proteasome for degradation^13,14^. Notably, the overexpression of Cuz1 decreases cell viability under normal growth conditions^14^.

In *C. elegans*, one ZFAND gene, arsenite-inducible protein-1 (AIP-1), is a homolog of the mammalian gene AIRAP^11^. Deletion of the AIP-1 gene increases sensitivity to arsenite, which is rescued by the increased level of AIP-1 under its own promoter^11,12,30^. An interesting feature of AIP-1 is that it appears to be associated with the ER membrane by its C-terminal CAAX motif for normal lifespan, which is also present in its mammalian homolog, AIRAP-like (AIRAPL)^30^. However, membrane association is not necessary for its protective function against arsenite.

For *Drosophila*, FlyBase lists four genes (CG12795 (2B), CG30094, CG15368 and Doctor No (Drn)) with the AN1 sequence, and the structures for both proteins (AF-Q9W371-F1 for CG15368 and AF-Q9VHF4-F1 for Drn) predicted by AlphaFold show AN1-type Zn fingers. Compared with other genes, Drn also has an A20 Zn finger at the N-terminus, which resembles AWP1/ZFAND6^52^. Drn is a component of the JAK/STAT pathway and regulates the endocytic trafficking of Dome, the receptor of the pathway. The roles of the Zn finger domains in Drn and the functions of genes are not well characterized.

In plants, gene family size seems to be greater than those in other kingdoms, e.g., 14 genes in *Arabidopsis thaliana*, 19 genes in *Populus trichocarpa*, 17 genes in *Oryza sativa* and 20 genes in *Ipomoea batatas*^53–55^. As many gene names suggest, i.e., stress-associated proteins (SAPs), their functions involve enhanced adaptive responses to various extra- and intracellular stresses. Accordingly, some genes (e.g., AtSAP5, OSISAP1 and Pha13) are inducible upon stresses such as salt, drought, heat, cold, and heavy metals^53,56,57^. For example, Pha13 is inducible by viral infection in orchids, which enhances antiviral immunity, and the overexpression of its Arabidopsis homolog AtSAP5 improves tolerance to dehydration^54,56^. Intriguingly, an in vitro ubiquitination assay revealed that the AtSAP5 AN1 domain and Pha13 A20 domain have ubiquitin ligase activity, i.e., autoubiquitination, although whether their E3 activities contribute to increased tolerance to the examined stresses is not understood^54,56^. Sequence comparison revealed that these two genes are homologs of OSISAP1, ZFAND5, and ZFAND6^54^; however, their ubiquitin ligase activities have not yet been reported in other ZFAND proteins. Notably, the robust protective effects of ZFAND proteins on the adaptation of plant physiologies to various stresses likely imply that the functions of ZFAND proteins in other eukaryotes also go beyond proteostasis and likely play roles in the regulation of diverse physiologies. Thus, it is very likely that ZFAND proteins in other eukaryotes influence broader cellular processes than is known.

### Mammalian ZFAND proteins

The mRNA or protein levels of mammalian ZFANDs are frequently upregulated, particularly in certain cancer cells, e.g., colon, papillary thyroid, and pituitary neuroendocrine tumors, or upon exposure to certain environmental toxic agents^11,58–61^.

ZFAND1 appears to be a functionally conserved homolog of Cuz1 and AIRAPL, particularly in response to arsenite treatment^14,62^. Exposure to arsenite results in the formation of stress granules (SGs), and ZFAND1 colocalizes with these foci^62^. Like Cuz1 and AIRAPL, ZFAND1 interacts with the proteasome and p97/cdc48 upon arsenite treatment, suggesting its roles in the recruitment of the proteasome and p97 to SGs and clearance of the granules^62^. Like Cuz1, ZFAND1 differs from other ZFAND proteins in that it contains a UBL domain, which is required for the clearance of stress granules. However, it remains unknown whether this function of UBL is mediated by the interaction of ZFAND1 with the proteasome or p97. Pulldown assays and methyl NMR spectrometry suggested that ZFAND1’s UBL is involved in the interaction with the N-domain of p97^52^. However, it remains unclear why one ZFAND1 binds one of the six N-domains of the p97 homohexamer or if the binding of cofactors, i.e., UFD1 and NPL4, determines the position of ZFAND5.

ZFAND2A and ZFAND2B have been studied relatively more than other family proteins and are also known as AIRAP and AIRAPL, respectively. Whereas AIRAPL expression is constitutive, AIRAP levels increase in response to certain proteotoxic stresses, such as heat shock, proteasome inhibition or arsenite treatment, in which the HSF1-HSF2 complex binds to its promoter, although its induction upon heat shock was not observed in other studies^11,63^. Moreover, although the association of AIRAPL with the proteasome increases upon arsenite treatment and the worm homolog AIP-1 is required for resistance to arsenite^30^, Turakhiya et al. reported that AIRAPL function is not involved in the clearance of stress granules induced by arsenite^62^. As mentioned above, the presence of the membrane binding motif suggests that AIRAPL associates with membranous compartments, and transiently transfected AIRAPL colocalizes with the ER membrane^30^. Among the relevant interesting questions is whether this membrane association is constitutive or regulated by certain stresses. In relation to its possible roles in the ERAD pathway, AIRAPL interacts with the VCP/p97 complex with its cofactors (i.e., UBXD8/FAF2, NPL4, and UFD1) on the ER membrane and regulates the translocation of ERAD- and non-ERAD-secreted proteins, which is dependent on the signal peptide^16^. Furthermore, mice lacking AIRAPL exhibited myeloproliferative neoplastic phenotypes, e.g., abdominal distension, wasting, and eventually premature death, revealing role of AIRAPL in suppressing myeloid transformation. This tumor suppressor function results from the ubiquitination and degradation of insulin-like growth factor 1 receptor (IGF1R) promoted by AIRAPL and requires key players in ERAD, such as p97, FAF2 and BAG6^64^. These intriguing findings support the significant implications of AIRAPL for understanding proteinopathies. Further studies should investigate how AIRAP and AIRAPL contribute to the ERAD pathway, specifically the regulatory mechanism for the proteasome and p97 complexes.

ZFAND3 contains an N-terminal A20 domain and a C-terminal AN1 domain connected by a flexible region, as observed in ZFAND5 and ZFAND6. ZFAND3 was initially reported as Tex27 from subtractive library screening during spermatogenesis, and its expression is differentially upregulated in the testis during the haploid stage of spermatogenesis^65^. ZFAND3 expression is also high in hepatocytes in a diabetic mouse model, and an increase in ZFAND3 levels improves glucose metabolism in high fat-diet-fed mice^66^. RNAi screening for factors involved in the invasive capacity of glioblastoma revealed that ZFAND3 is a transcriptional regulator that controls genes linked to glioblastoma cell invasion in vitro and in vivo^58^. Interestingly, when overexpressed, ZFAND3 accumulates in the nucleus and increases the invasive capacity of noninvasive glioblastoma stem-like cells (GBMSCs), demonstrating the tumorigenic potential of ZFAND3. In GBMSCs expressing epitope-tagged ZFAND3, a few proteasome subunits are coimmunoprecipitated, which may suggest the involvement of proteasome activity in the invasive capacity of glioblastoma. Thus, related questions include whether the proteolytic capacity of proteasomes contributes positively or negatively to invasiveness. In addition, whether the proteasome enhances or inhibits ZFAND3 transcriptional activity or whether an independent pathway is involved is unknown.

ZFAND4, also known as ANUBL1, shares high amino acid sequence identity, but ZFAND4 is 74 amino acids longer at the N-terminus. It is reported to be upregulated in certain cancers, e.g., gastric cancer or oral squamous cell carcinoma, suggesting that it is an oncogene or marker^59,60^.

ZFAND5, also known as ZNF216, was initially reported to be expressed in the cochlea from the study of autosomal recessive nonsyndromic hearing loss, although it is not involved in deafness^67^. As increased mRNA levels of ZFAND3 and ZFAND4 have been reported in certain cancer cells, the ZFAND5 mRNA level was also observed to be high in the cells of perihilar cholangiocarcinoma, colon cancer, medulloblastomas and metastatic nasopharyngeal carcinomas but lower in hepatocarcinoma cells^68^. The differential expression of these genes suggests a possible correlation between ZFAND5 levels and tumorigenesis. Moreover, the basal level of ZFAND5 protein also differs between cell types; for example, it is higher in the mouse brain and heart than in the liver and skeletal muscle (e.g., tibialis anterior)^28^. When the stability of ZFAND5 was measured in myoblasts with relatively high ZFAND5 contents, its half-life was found to be short, approximately 2.5 h^28^. Interestingly, an unpublished study that its level is low in a ubiquitin-independent manner (see below). Like ZFAND2A, ZFAND5 is induced by several stimuli, but regulatory factors, such as the nature of the signaling pathway and transcription factors that respond to various stimuli, have not been reported (also see below). Notably, siRNA screening revealed that ZFAND5 is involved in the degradation of two missense mutants of dystrophin found in Duchenne or Becker muscular dystrophy, highlighting its role in the hydrolysis of specific proteins in skeletal muscle in addition to the overall proteolysis shown in muscle atrophy^15,69^.

ZFAND6, also called AWP1, is expressed ubiquitously^70^. Its domain organization resembles those of ZFAND3 and ZFAND5. Like ZFAND3, ZFAND4 and ZFAND5, ZFAND6 have also been reported to be a potent inhibitor of NF-kB activation*;* however, it also seems to be required for NF-kB activity and inhibits tumor necrosis factor alpha (TNFα)-induced apoptosis, whereas its overexpression inhibits NF-kB activation^71,72^. The *Drosophila* ortholog Doctor No (Drn) stimulates the JAK/STAT pathway by binding to ubiquitinated receptors of ligands and facilitating the endocytic trafficking of the receptors^52^. Another interesting function of ZFAND6 includes its interaction with monoubiquitinated Pex5 through its A20 domain and its export from the peroxisomal matrix^73^. This process requires Pex1-Pex6, a peroxisomal AAA ATPase complex, and both A20 and AN1 Zn fingers are required for binding to Pex6 ATPase. In *Xenopus*, ZFAND6 plays an essential role in the development the neural crest and pigmentation^74^. GWAS identified the ZFAND6 susceptibility gene for type 2 diabetes and suggested correlations with insulin secretion and beta*-*cell function^75–77^. It will be interesting to explore whether this role is associated with its regulatory role in peroxisomal protein export.

ZFAND7, also known as immunoglobulin mu (μ) DNA-binding protein 2 (IGHMBP2), appears to be the only mammalian family member that has enzymatic activity and is a DNA/RNA helicase involved in the control of transcription or splicing^78^. In particular, mutations in this gene are associated with autosomal recessive degeneration of motor neurons, manifested as weakness of the distal limb and spinal muscular atrophy with respiratory distress type 1 (SMARD1)^79^. In mice, a spontaneous recessive mutation in neuromuscular junctions (known as Ighmbp2^nmd-2J^) causes progressive atrophy of limb muscles, but IGHMBP2 transgenic mice specific to neurons show rescued phenotypes of motor neuron degeneration, demonstrating a causative role in the progression of disease^79,80^. However, transgenic animal lines develop cardiac and skeletal myopathy, which suggests that IGHMBP2 is involved in the maintenance of differentiated tissues, although the pathogenic mechanism for such phenotypes resulting from IGHMBP2 alteration remains unclear^80^. Another disease associated with IGHMBP2 mutations is Charcot-Marie-Tooth disease type 2 (CMT2)^81^. Although specific mutations have been reported to be correlated in patients with CMT2, their causative roles and whether the AN1 domain plays any role in pathogenesis are not fully understood.

### Regulation of ZFAND5 level when its expression is induced or normal

Western blotting has shown that the levels of ZFAND5 in cell lines (e.g., HEK293, HeLa, C2C12 myoblast and MM.1 s) and some mouse tissues (e.g., liver and skeletal muscles) under normal growth conditions are very low^28^. Nondetectable levels might have resulted from issues with antibody sensitivity, but it is also possible that either the gene is not transcribed or the translated protein is rapidly hydrolyzed by an unknown mechanism. An unpublished study revealed that proteasome inhibition increases the amount of ZFAND5 detected by Western blot in human multiple myeloma cell lines, indicating that the basal level of ZFAND5 in HEK293 and HeLa cells is maintained at a low level due to rapid proteasomal degradation (unpublished data). In contrast to cells containing low levels of ZFAND5, the questions of why and how some cells, such as those in the brain and heart, contain high ZFAND5 levels are also interesting, especially if higher ZFAND5 plays a regulatory role in proteostasis in those cells.

Intriguingly, in unpublished studies, to determine whether the basal level of ZFAND5 is controlled by ubiquitin-dependent degradation, ubiquitination was blocked by the inhibition of the ubiquitin-activating enzyme UBA1. Surprisingly, mass spectrometry analysis revealed that the basal level of ZFAND5 is not upregulated but even further decreased (unpublished data), which suggests, together with the proteasome inhibition results, that the basal level of ZFAND5 is maintained at a low level through ubiquitin-independent degradation by the proteasome. However, in a purified system, the 26S or 20S proteasome does not degrade ZFAND5, suggesting that an additional factor or posttranslational modification is necessary^29^. Thus, investigating the degradation mechanisms for basal and induced levels of ZFAND5 will provide important examples and new insights into understanding ubiquitin-dependent and ubiquitin-independent proteolysis by the 26S and 20S proteasomes.

The questions related to the degradation of ZFAND5 and possibly its functions involve how the proteasome recognizes and selects nonubiquitinated ZFAND5 proteins in cells. Is there a factor that mediates the recognition of ZFNAD5 by the proteasome? As a recent elegant study demonstrated that midnolin mediates the degradation of ubiquitin-independent proteolysis^82^, it will be interesting to examine the possible role of midnolin in the ubiquitin-independent degradation of ZFAND5. As an alternative, not exclusive to other possibilities, is there any posttranslational modification of ZFAND5 involved in its recognition by the proteasome? Other forms of proteasome complexes are known to be involved in ubiquitin-independent degradation. Do the 26S proteasome or other complexes containing PA200 and PA28αβ degrade ZFAND5? Furthermore, is p97/VCP activity required for unfolding ZFAND5 before proteasomal degradation?

Like ZFAND2A, ZFAND5 is also inducible in response to various stimuli. In macrophage-derived (RAW 264.7) and HEK293 cells, the mRNA and protein levels of ZFAND5 increase in response to proinflammatory cytokines (e.g., TNF-α, IL-1β, RANKL, and LPS) and the PKC activator 12-O-tetradecanoylphorbol-13-acetate (TPA)^83,84^. In atrophying skeletal muscle by denervation, starvation or high glucocorticoid treatment, the ZFAND5 content also increases, which contributes to muscle wasting^15^. As an inducible gene, its content must be degraded. Immunoprecipitation followed by mass spectrometry revealed that ZFAND5 was elevated upon treatment with TNF-α, and IFN-γ was enriched inside the 20S proteasome chamber 1 h after cytokine stimulation^84^. To eliminate the induced ZFAND5 protein specifically after stimuli are attenuated, the selection of such an induced substrate by ubiquitination should ensure specific degradation. Nevertheless, whether the ubiquitination of induced ZFAND5 is required for its proteasomal degradation remains to be determined. If proteolysis is ubiquitin dependent, identification of the responsible ubiquitin ligase will be important for understanding ZFAND5 functions and suggesting a new therapeutic intervention for muscle wasting.

## ZFAND5 as a 26S proteasome activator and shuttling factor for proteasomal degradation

Our recent efforts on ZFAND5 demonstrated the first biochemical evidence for how a shuttling factor accelerates the hydrolysis of ubiquitinated substrates by the 26S proteasome^29^. Like other UBL-UBA proteins, ZFAND5 also associates with both ubiquitin conjugates and the 26S proteasome, which suggests that ZFAND5 may stimulate the recruitment of substrates to the proteasome^15,28,29^. In addition to NMR and crystal structure studies, assays measuring the degradation of ubiquitinated proteins have confirmed ZFAND5 A20 as the ubiquitin-binding domain essential for substrate degradation in vitro and in vivo^28,29^. Given the essential role of the ZFAND5 AN1 domain in stimulating proteasomal peptidase activity, the binding of the proteasome subunit to the AN1 domain was investigated, and cross-linking followed by mass spectrometry revealed the specific interaction of the AN1 domain with Rpt5^28,29^. The cryo-EM structure revealed that the AN1 Zn finger is located between the small and large subdomains of the ATPase domain of Rpt5, whereas the A20 domain was not found in the structure because of the flexible region between A20 and AN1^29^. However, the flexible region likely allows the A20 domain to move freely to find and interact with the ubiquitinated substrate and perhaps hand over the substrate to one of the intrinsic ubiquitin receptors, especially those close to the Rpt5 ATPase, Rpn1 and Rpn10 or even directly to the translocation channel.

Interestingly, another important feature of ZFAND5 A20 is its binding to the polar surface of ubiquitin around Thr55 and Asp58, unlike most ubiquitin-binding domains, which bind to the hydrophobic β-sheet patch around Ile44^85^. Thus, this feature enables ZFAND5 to keep the hydrophobic patch of the same ubiquitin on the substrate available for binding to Rpn1 or Rpn10. Alternatively, one of the ubiquitins on the substrate may be held by ZFAND5 A20, and another ubiquitin in the conjugates may interact with the Rpn1 T1 or T2 site or Rpn10 UIM. This ability of flexible ZFAND5 to bind ubiquitin may support the function of ZFAND5 as either a shuttling factor or a proteasome activator once it is on the 26S proteasome to eventually facilitate substrate hydrolysis, although the possible role of the distinct ubiquitin interaction of A20 has not been examined experimentally.

Binding of the AN1 Rpt5 ATPase domain causes several significant changes in the 19S structure, especially widening the channel of the OB ring from the outer surface to the gate of the 20S proteasome, which should facilitate deubiquitination, substrate unfolding and degradation in the 20S complex^29^. Another key feature of ZFAND5-mediated 26S proteasome activation revealed by the single-molecule assay is increased substrate binding to 26S^29^. Because one ZFAND5 interacts with the 26S complex, it provides just one more ubiquitin-binding domain, A20, in addition to three 19S subunits, i.e., Rpn1, Rpn10 and Rpn13, for recognizing ubiquitin. Moreover, the median interaction time of ZFAND5 with 26S is approximately 1.3 s. Thus, the addition of one more ubiquitin-interacting domain cannot fully explain the activation of several activities of the proteasome, such as increases in ATPase activity, peptidase activity, Rpn11 deubiquitinase activity, increased substrate dwell time on 26S and degradation. Thus, to achieve all the activation features induced by ZFAND5, the conformational changes induced by AN1 binding and even the absence of ZFAND5 on 26S (e.g., Z-_**C**_ state^29^,) likely make 26S more prepared (preferred structure) for substrate interactions and degradation.

Intriguingly, the stimulatory effect of ZFAND5 on the degradation of ubiquitinated proteins requires the interactions of four residues with Rpn1 and Rpt1^29^. Inhibition of the interactions of ZFAND5 residues by point mutations abolishes stimulated protein degradation; however, the degradation levels of ubiquitinated proteins and short peptides by ZFAND5 mutation remain the same as the levels of degradation by the 26S proteasome alone. Thus, the inhibition of ZFAND5-mediated 26S activation specifically suppresses stimulation but does not block basal proteasome activity, unlike the inhibition of the catalytic sites of the 20S complex by known proteasome inhibitors. Moreover, stimulation of proteasomal degradation was also observed with the synthesized peptide of 19 amino acids in ZFAND5’s C-terminus, which bears the four residues critical for interactions with Rpn1 and Rpt1. These findings suggest the possibility of developing both 26S proteasome inhibitors with few or no harmful effects on normal cell viability and small-molecule activators.

## Possible activation mechanisms by which ZFAND proteins regulate proteolysis

**Fig. 1:**
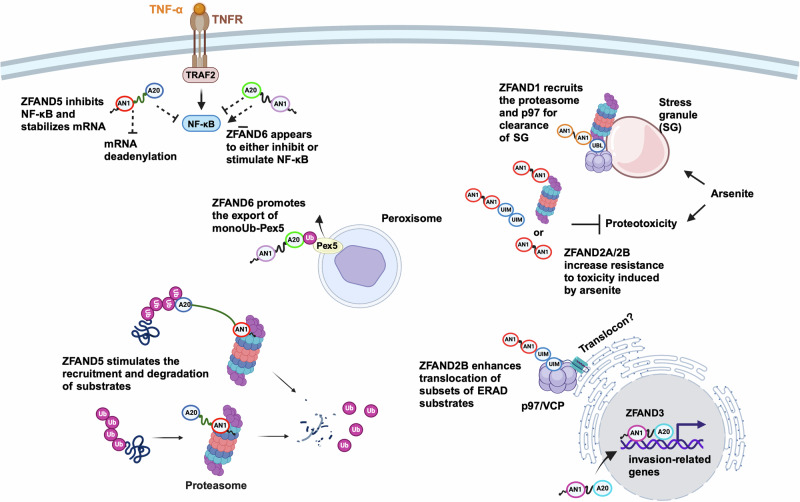
Roles of ZFAND proteins in the regulation of cell physiology. The schematic presents a simplified overview of the primary functions of ZFAND proteins in mammalian cells. Although all ZFAND proteins contain one or two AN1 domains, they appear to play different regulatory roles in various cellular processes. ZFAND1 can recruit p97 and the proteasome to facilitate clearance of the stress granule formed by arsenite. ZFAND2A levels are inducible by arsenite and interact with the proteasome, likely protecting cells against proteotoxicity. However, it is also possible that ZFAND2A functions independently or that other unknown regulators are involved in addressing proteotoxicity. ZFAND2B interacts with the proteasome upon arsenite treatment, which should also contribute to cellular resistance to arsenite-induced toxicity. Unlike other known ZFAND proteins, ZFAND3 is reported to translocate to the nucleus and appears to act as a transcriptional activator for genes involved in the invasion of glioblastoma. ZFAND5 appears to stimulate both the binding and degradation of ubiquitinated substrates by the 26S proteasome, whereas ZFAND6, with a similar domain array, interacts with hexameric Pex6 to facilitate the export of Pex5 from the peroxisome. Interestingly, both ZFAND5 and ZFAND6 inhibit NF-κB activity, although ZFAND6 is also known to stimulate it. Additionally, ZFAND5 regulates mRNA stability by inhibiting deadenylation. ZFAND proteins in other species are not shown here due to space limitations. The dotted lines indicate that the actions may be indirect.

Although some of their potential roles in regulating protein degradation and aggregation have been reported, the number of studies on ZFAND proteins remains insufficient to suggest a common regulatory mechanism. As a result, our understanding of how ZFAND proteins contribute to both the global and specific regulation of protein homeostasis is still very limited. For example, the biological functions of the AN1 domains within these proteins are largely unclear. Nevertheless, a summary schematic (Fig. 1) is presented to illustrate these potential roles (Fig. 1). ZFAND proteins that contain Ub-binding domains (such as UIM and A20), including ZFAND2B, ZFAND5, and ZFAND6, are likely involved in recruiting or facilitating the binding of target proteins to destinations such as the 26S proteasome for enhanced degradation, directing proteins to p97/VCP for unfolding or peroxisomes to facilitate the export of the peroxisomal protein Pex5. Despite its role in cellular adaptation to proteotoxicity, ZFAND2A lacks a Ub-binding domain. However, although ZFAND2A may not directly process ubiquitinated proteins, it could still promote Ub-independent hydrolysis by the 26S proteasome or enhance protein unfolding via p97. Additionally, the critical roles of UBL in ZFAND1 and CUZ1 suggest more diverse regulatory roles of ZFAND proteins in protein unfolding under proteotoxic conditions. Other diverse functions of ZFAND proteins, including their roles in regulating the NF-κB and JAK/STAT pathways, will also benefit from further studies.

## Conclusions and remaining questions

Although the stimuli and conditions that induce muscle wasting, heat shock, cAMP signaling, and cGMP have been investigated extensively, their regulatory roles in proteasome activation have just begun to be investigated. The downstream effector proteins for directly stimulating proteasome activities have been demonstrated in several stimuli, e.g., ZFAND5 for muscle wasting, ZFAND2A upon arsenite treatment, and PKA and PKG in the cAMP and cGMP pathways, whereas the identity of such an effector for heat shock remains unknown^12,20,22,25,29^. Notably, two ZFAND proteins, ZFAND1 and ZFAND2B appear to control the function of p97 in the clearance of stress granules and the translocation of a nascent polypeptide, which may stimulate further studies related to proteinopathies^16,62^. In addition to these stimuli having broad physiological significance, other conditions and unidentified factors may be unexplored in this regard.

It is likely that the shuttling factors add other layers of regulation, i.e., activation to proteasomal degradation, probably in different manners than ZFAND5 does, which remains to be investigated. The following are among the remaining questions. Do the shuttling factors use the same or similar mechanisms as ZFAND5 to enhance substrate delivery to 26S and subsequent proteolysis? What are the distinct roles of several shuttling factors in the delivery of substrates? Perhaps a related question is as follows: what are the distinct clients for them? Thus, how do the different shuttling factors recognize different clients? In addition to the complex regulation of proteasomes, e.g., intrinsic enzymatic activities, many interacting regulators, substoichiometric subunits, posttranslational modifications and the coordination of all these factors, it is very likely that there are more unidentified proteasome activators under known and less characterized conditions. Thus, further studies on proteasome activation under specific conditions will provide novel insights into our understanding of proteasomal degradation and may lead to unexpected and therapeutic applications in related diseases.
